# An overview of the molecular and epidemiological features of HIV-1 infection in two major cities of Bahia state, Brazil

**DOI:** 10.1590/0074-02760160458

**Published:** 2017-06

**Authors:** Amanda GM Amaral, Isabele B Oliveira, Diego C Carneiro, Luiz CJ Alcantara, Joana P Monteiro-Cunha

**Affiliations:** 1 Universidade Federal da Bahia, Departamento de Biofunção, Núcleo de Bioinformática, Salvador, BA, Brasil; 2 Fundação Oswaldo Cruz-Fiocruz, Instituto Gonçalo Moniz, Salvador, BA, Brasil; 3 Fundação Bahiana para o Desenvolvimento das Ciências, Escola Bahiana de Medicina e Saúde Pública, Salvador, BA, Brasil

**Keywords:** HIV subtype, drug resistance, Bahia, genotyping, mutations, epidemiology

## Abstract

**BACKGROUND:**

The high mutation rate of the human immunodeficiency virus (HIV) has created a public health challenge because the use of antiretroviral drugs can generate selective pressure that drives resistance in these viruses.

**OBJECTIVE:**

The aim of this work was to characterise the molecular and epidemiological profile of HIV in Bahia, Brazil.

**METHODS:**

DNA sequences from regions of HIV *gag*, *pol,* and *env* genes were obtained from previous studies performed in this area between 2002 and 2012. Their genotype and drug-resistance mutations were identified using bioinformatics tools. Clinical and epidemiological data were analysed.

**FINDINGS:**

Among 263 individuals (46.4% male), 97.5% were asymptomatic and 49.1% were receiving treatment. Most of the individuals were 31 to 40 years old (36.9%) and infected through heterosexual contact (40.7%). The predominant genotype was B (68.1%) followed by BF recombinants (18.6%). Among the individuals infected with either F or BF genotypes, 68.4% were women and 76.8% were infected through heterosexual transmission. The prevalence of associated mutations conferring antiretroviral resistance was 14.2%, with 3.8% of all mutations conferring resistance to protease inhibitors, 9.43% to nucleoside reverse transcriptase inhibitors, and 8.5% to non-nucleoside reverse transcriptase inhibitors. Drug resistance was higher in individuals receiving treatment (26.1%) than in the drug-naïve (4.3%) individuals.

**MAIN CONCLUSIONS:**

This study will contribute to the understanding and monitoring of HIV epidemic in this Brazilian region.

Antiretroviral drugs (ARVs) are used to treat the human immunodeficiency virus (HIV), which causes acquired immune deficiency syndrome (AIDS). ARVs minimise the symptoms and viral load of HIV, especially if it is diagnosed and treated quickly. The HIV diagnostic test and antiretroviral therapy are free of charge in Brazil. Despite improvements brought about by ARVs in the quality of life and life expectancy of infected individuals, HIV/AIDS is still considered one of the most severe epidemics in the world. This is because of the absence of an effective preventive vaccine for the virus, which leads to constantly increasing numbers of infected people (UNAIDS 2014). Despite the high incidence of the disease, the mortality of AIDS is declining (MS 2014). Currently, although it is possible to live a normal life without symptoms of the disease through ARV treatment, it is still necessary to control viral dissemination owing to the absence of appropriate prophylaxis.

HIV has a high mutation rate, which can be correlated with the emergence of drug-resistant variants when viral replication is not sufficiently inhibited (Chen et al. 2005). This is why it is so difficult to develop efficient prophylactic methods and lasting treatments with the same class of drugs. In addition, HIV has significant genomic diversity, and recombination between different viral strains is quite common. This genetic variability creates a complex global pattern in the distribution of viral subtypes. In Brazil, the predominant genotype is B, followed by BF recombinants, and then F and C subtypes (Monteiro-Cunha et al. 2011). However, since it is a large country, the distribution model of viral subtypes is distinct for each Brazilian region (Morgado et al. 1994, 1998, Couto-Fernández et al. 2005, Inocêncio et al. 2009, Monteiro et al. 2009, Arruda et al. 2011, Costa et al. 2013). In Bahia, which is the largest state in north-eastern Brazil, 26,268 cases of HIV infection had been catalogued by October 2015 (SUVISA 2015). Previous studies conducted in the capital, Salvador (Monteiro et al. 2009, Araújo et al. 2010, Santos et al. 2011), and in an inner city, Feira de Santana (Santos et al. 2009, Monteiro-Cunha et al. 2011), found that the prevalence of subtypes in this region was similar to the national distribution.

To better characterise the HIV/AIDS epidemic in Bahia, the present study used clinical and epidemiological data from HIV-1 infected individuals recruited between 2002 and 2012 to determine population and viral features. This analysis will contribute to the understanding and monitoring of the dissemination of HIV in this Brazilian region.

## MATERIALS AND METHODS

Clinical, epidemiological, and molecular data generated in previous studies from our group in Salvador and Feira de Santana were collected and organised in a database. Thirty-two patient samples were obtained in the Monteiro et al. (2009) study. From these, 32 *gag* sequences and 31 *env* sequences were included in the present study. Fifty-eight patient samples were obtained in the Araújo et al. (2010) study, from which 42 *gag* sequences and 40 *env* sequences were included in the present study. Santos et al. (2011) collected 57 samples, and their *pol* sequences are included in this study. Thirty-nine of the individuals were analysed in both the Araujo and Santos studies. Monteiro-Cunha et al. (2011) analysed a cohort of 58 individuals (50 women and eight children), from which *pol* sequences were included in this work. Monteiro-Cunha et al. (unpublished observations) also studied a cohort of 97 patients aged 18 years or older who were HIV-1 positive and treatment-naïve, from which *pol* sequences were included in the present analysis. Patients received regular follow-up consultations at the Professor Edgard Santos University Hospital, or at the Specialised Centre for Diagnosis, Care and Research in AIDS, STDs and Viral Hepatitis (CEDAP), and signed a term of informed consent to participate in this study, which was approved by the Oswaldo Cruz Foundation (FIOCRUZ) Institutional Review Board (protocol number 376/2012). The blood samples were collected during 2012 and sent to the Laboratory of Haematology, Genetics and Computational Biology/IGM/FIOCRUZ for processing. Clinical and epidemiological data were obtained from patient medical records and interviews. Genomic DNA was extracted using a QIAamp DNA Blood Mini Kit (QIAGEN, Valencia, CA). Protease and reverse transcriptase (RT) regions of the *pol* gene were amplified and sequenced as previously described (Sucupira et al. 2007). The generated sequences are available in the GenBank database (https://www.ncbi.nlm.nih.gov/genbank/), under the accession numbers KT950963-KT951059.

**Fig. 1:**
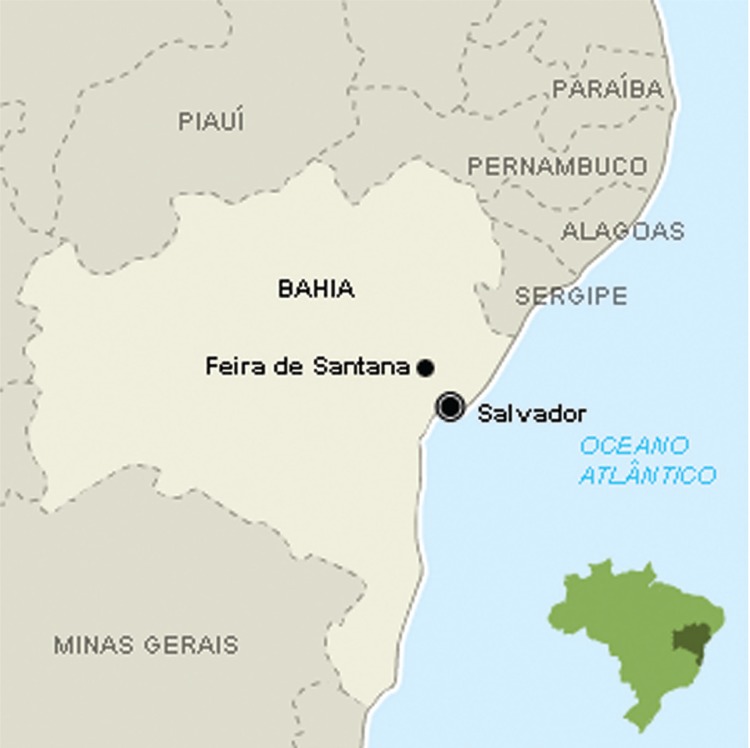
: Salvador and Feira de Santana are the two most populous cities in Bahia state located in North-eastern Brazil.

Overall, the 263 HIV-1-seropositive patients who were analysed were recruited between 2002 and 2012 in Salvador and Feira de Santana. With around three million people, Salvador it is the largest city in the North-east Region and the 3rd-largest in the country, after São Paulo and Rio de Janeiro. Salvador also accounts for around 60% of the AIDS cases reported in Bahia (SUVISA 2015). Feira de Santana is the second-most populous city in the state, with a population of around 600,000. It is located 100 km northwest of Salvador. Being a major junction of north-eastern Brazil’s highways nearby, Feira de Santana functions as a crossroads for the traffic coming from the south and west-central portions of Brazil bound for Salvador and other important cities of the north-east. Therefore, Feira de Santana is now an important and diverse commercial and industrial centre of north-eastern Brazil (Fig. 1) and is the city with the second largest number of AIDS cases in the state, accounting for nearly 7% of total AIDS cases (SUVISA 2015).

All nucleotide sequences from *gag*, *pol*, and *env* were obtained by polymerase chain reaction (PCR) and direct sequencing of genomic DNA extracted from blood samples collected from the cohorts of previous studies. Sequences were aligned in fasta file format, using BioEdit software (Hall 1999). The *gag*, *pol*, and *env* genomic regions analysed had nucleotide positions 896-1970, 2318-3161, and 6816-7668 relative to the HXB2 reference genome, respectively.

The viral subtype was confirmed using the HIV REGA subtyping tool (http://www.bioafrica.net/). Mutations associated with drug resistance were identified using the current version of the Stanford Database (STdb) program [http://hivdb.stanford.edu/] version 8.1.1, which was launched in September 2016 and presents an updated list of mutations.

GraphPad Prism (Version 7, GraphPad Software Inc., CA, USA) was used to perform statistical analysis by Fisher’s exact test (two-tailed) to examine the significance of the association between two kinds of classification. P-values ≤ 0.05 were considered significant.

*Ethics* - The studies were approved by Oswaldo Cruz Foundation (FIOCRUZ) Ethics Committee and all the included individuals signed an agreement to participate at the study.

## RESULTS

This study analysed HIV gene sequences in 263 HIV-1-seropositive patients, out of which 74 samples had at least two sequenced regions (*gag* + *env*, *gag* + *pol*, *pol* + *env*, or *gag* + *env* + *pol*), and the other 189 had only one (*gag*, *env*, or *pol*). When more than one genomic region was available, the genotypes were combined to determine the final genotype.

**Fig. 2:**
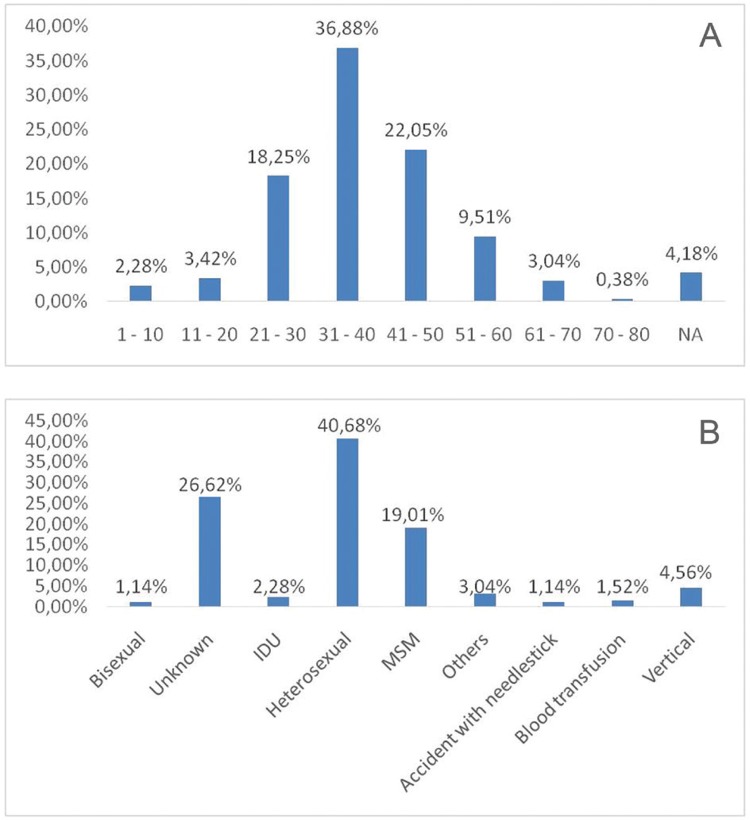
: (A) characterisation of the human immunodeficiency virus (HIV)-1 infected population age group in Bahia state; (B) distribution of the transmission type in the HIV-1 infected population in the Bahia state.

The epidemiological and molecular characteristics of patients analysed in this study are shown in Table I. Following genotyping analysis, 179 (68.1%) samples were found to be subtype B, five (1.9%) were subtype C, and 23 (8.7%) were subtype F. The study also observed intergenic BF recombinant viruses in six (2.3%) individuals, intragenic (BFi) recombinants in 49 (18.6%) individuals, and DF recombination in one individual (0.4%). One hundred and twenty-nine (49.0%) individuals were adhering to a therapeutic regimen, whereas 118 (44.9%) were treatment-naïve. For 16 patients (6.1%) this information was not available. One hundred and thirty-two samples (50.2%) were obtained from women, 122 (46.4%) were obtained from men, and eight (3.0%) were from children of undetermined gender. One adult individual did not have available gender information. The ratio of male to female in this study was therefore 0.92:1. To correct for selection bias, we excluded the cohort comprised exclusively of women and children (Monteiro-Cunha et al. 2011) from subsequent gender ratio calculations, which changed the total gender ratio to 1.47:1. The gender ratio for subtype B was 2.11:1, whereas the ratio for the group formed by F-related viruses (pure subtype F and BF recombinants) was 0.67:1. The age variation within this population and the transmission mode prevalence are shown in Fig. 2A. The mean age was 36 years, and the range was 2-73 years. The main transmission mode was heterosexual contact, which was reported by 40.7% of the participants, followed by men who have sex with men (MSM) (19.0%) (Fig. 2B). One hundred and forty-five patients did not have their clinical status defined, and from the remaining population of 118 individuals, 115 (97.5%) were asymptomatic, and three (2.5%) were symptomatic.

**TABLE I:** Clinical, molecular and epidemiological characteristics of human immunodeficiency virus (HIV)-1-infected individuals in Bahia state

Characteristics (n = 263)	
Age (years)	
Minimum	2
Median	36
Mean	36.5
Maxim	73
Gender (%)	
Male	122 (46.39%)
Female	132 (50.19%)
Child	08 (3.04%)
NA	01 (0.38%)
Year of HIV-1 diagnosis	
1991 - 1996	8 (3.04%)
1997 - 2001	20 (7.6%)
2002 - 2006	20 (7.6%)
2007 - 2012	85 (32.32%)
NA	130 (49.43%)
Exposure category	
Heterosexual	107 (40.68%)
MSM	50 (19.01%)
Bisexual	03 (1.14%)
IDU	06 (2.28%)
Accident with needlestick	03 (1.14%)
Vertical	12 (4.56%)
Blood transfusion	4 (1.52%)
Others	08 (3.04%)
NA	70 (26.62%)
Treatment	
Treated	129 (49.05%)
Naïve	118 (44.87%)
NA	16 (6.08%)
Clinical state	
Symptomatic	03 (1.14%)
Asymptomatic	115 (43.72%)
NA	145 (55.13%)
Genotype	
B	179 (68.1%)
BF	06 (2.3%)
BFi	49 (18.6%)
C	05 (1.9%)
DF	01 (0.4%)
F	23 (8.7%)

IDU: intravenous drug use; MSM: men who have sex with men; NA: not available.

The subtype prevalence was compared between the different populations included in the present work in order to investigate temporal trends in the subtype distribution (Table II). The earlier studies (Monteiro et al. 2009, Araújo et al. 2010) used the *env* and *gag* regions for subtyping. When comparing these two data sets, we observed an increase in the prevalence of pure subtypes B (84.0-91.8%) and F (2.3-4.9%) and a decrease in BF the prevalence of recombinants (13.1-3.3%) between 2002 and 2006. The most recent studies used the HIV *pol* regions for subtyping. When comparing the data from Santos et al. (2011) who collected samples in 2006 with unpublished data from 2012, we observed a decrease in the prevalence of subtype B (77.2-73.2%) and in the prevalence of BF recombinants (21.0-8.3%) from 2006-2012 in Salvador city, while subtype F prevalence has increased (1.8-14.4%). These trends were similar when considering only the heterosexually infected individuals in these two populations.

**TABLE II:** Subtype prevalence among infected individuals in Salvador and Feira de Santana (Bahia, Brazil) between 2002 and 2012

Sampling year (publication)	Subtype prevalence (%)				

	B	F	C	BF	FD
2002^*a,c*^ (Monteiro et al. 2009)	84%	2,3%		13,1%	0,6%
2006^*a,c*^ (Araújo et al. 2010)	91,8%	4,9%		3,3%	
2006^*b,c*^ (Santos et al. 2011)	77,2%	1,8%		21%	
2007^*b,d*^ (Monteiro-Cunha et al. 2011)	67,2%	6,9%	1,7	24,1%	
2012^*b,c*^ [Monteiro-Cunha et al. (unpublished observations)]	73,2%	14,4%	4,1	8,3%	

a: based on *env* and *gag* subtyping; b: based on *pol* subtyping; c: samples from Salvador; d: samples from Feira de Santana.

The protease and reverse transcriptase regions of the HIV *pol* gene in 212 patients were sequenced. One hundred and fifty-six sequences (73.6%) had subtype B, five (2.4%) had subtype C, and nineteen (9.0%) had subtype F. TBF recombination was observed in two (0.9%) samples and BFi was observed in 30 (14.2%) samples. ARV drug resistance mutations were then analysed for protease inhibitors (PIs), nucleoside reverse transcriptase inhibitors (NRTIs), and non-nucleoside reverse transcriptase inhibitors (NNRTIs).

A total of 196 (92.5%) viral strains were found to be susceptible to PIs, and from 16 sequences with mutations associated with any level of resistance to PIs, 12 (75.0%) were subtype B and four (25.0%) were subtype BFi. Four (25.0%) sequences had a major mutation to PI, whereas four (25.0%) had both major and minor mutations, and eight (50.0%) had only minor mutations. When considering only viruses that had major mutations, as recommended by the World Health Organization (WHO), the prevalence of mutations associated with resistance to PIs found in the cohort was 3.77% (Table III).

**TABLE III:** Comparison of human immunodeficiency virus (HIV) drug resistance prevalence in Bahia state

Drug Resistance	Pedroso et al. (2007) ^*a*^	Monteiro-Cunha et al. (2011) ^*b*^	Santos et al. (2011) ^*c*^	Soares et al. (2014) ^*a*^	Present study - ARV naïve	Present study - ARV experienced
N	132	58	57	47	118	92
NRTI	9.8%	15.5%	24.6%	10.6%	0.85%	20.65%
NNRTI	11.4%	6.9%	21.0%	4.26%	1.69%	16.30%
PI	5%	5.2%	7.0%	6.38%	1.69%	6.52%
Total	18.9%	17.2%	24.6%	17%	4.24%	26.09%

a: drug naïve individuals only; b: 82.8% of individuals were ARV experienced; c: 77.2% of individuals were ARV experienced; ARV: antiretroviral drugs; N: total number of patients analysed; NNRTI: non-nucleoside reverse transcriptase inhibitors; NRTI: nucleoside reverse transcriptase inhibitors; PI: protease inhibitor.

One hundred and eighty-two (85.9%) isolates were susceptible to NRTIs, and from the 30 sequences harbouring mutations associated with any resistance level, 25 (83.3%) had genotype B, two (6.7%) had the recombinant genotype BFi, and three (10.0%) had genotype F. Out of these, 20 (66.7%) viruses had high or intermediate levels of resistance to NRTIs, and 10 (33.3%) had only low levels of resistance. Thus, the prevalence of mutations associated with intermediate and high level resistance to NRTIs in this population was 9.4% (Table III).

One hundred and seventy-eight (84.0%) strains were susceptible to NNRTIs. Out of the 34 viruses with mutations associated with any resistance level to NNRTIs, 27 (79.4%) were subtype B, three (8.8%) were subtype BFi, three (8.8%) were subtype F, and one (2.9%) was subtype C. For NNRTIs, 18 (52.9%) had a high or intermediate resistance level, and 16 (47.1%) had a low resistance level. Therefore, the prevalence of mutations associated with intermediate and high resistance level against NNRTIs in this cohort was 8.5% (Table III). Eighty percent of the mutated viruses were classified as subtype B.

Overall, 30 individuals (14.2%) had mutations associated with significant drug resistance. Eight individuals (3.7%) had resistance mutations for NRTI, seven (3.3%) for NNRTI, and three (1.4%) for PI. One (0.5%) patient had resistance mutations for PI and NRTI, seven (3.3%) for NRTI and NNRTI, and four (1.9%) individuals had resistance to all three drug classes. The most prevalent mutation associated with resistance to PIs was V82A (1.9%), conferring resistance to Indinavir (IDV) and Lopinavir (LPV). The mutation I54V (1.4%) showed susceptibility only for Darunavir (DRV), and L90M (1.4%) only for DRV and Tipranavir (TPV). The most prevalent mutation associated with resistance to NRTIs was M184V (6.1%), which conferred high resistance to Lamivudine (3TC) and Emtricitabine (FTC) drugs. The K70R mutation (3.3%) conferred resistance to Zidovudine (AZT). M41L (4.7%) combined with T215Y (2.4%) also had high resistance to AZT, but this also conferred intermediate to high resistance to Stavudine (D4T). Where the L210W mutation also appears in this set, it potentiates high resistance to AZT and d4T. If it appears alone, it appears to confer intermediate to high resistance against Abacavir (ABC) and Tenofovir (TDF). The most prevalent mutation associated with resistance to NNRTIs in this dataset was K103N (2.8%), followed by G190A (1.9%) and Y181C (1.4%). All of these mutations conferred high or intermediate resistance against Nevirapine (NVP) and Efavirenz (EFV), and the latter also conferred intermediate resistance to Etravirine (ETR) and Rilpivirine (RPV). The prevalence of the other mutations ranged from 0.05% to 0.09% (Table IV).

**TABLE IV:** Molecular genotype and drug resistance profile of human immunodeficiency virus (HIV)-1-infected individuals living in Bahia state

City	Sample ID	Subtype	Treatment	NRTI	NNRTI	PI
SSA	BAS011	B	Yes		K103N	
	BAS015	BFi	Yes	K70R, M184V		
	BAS048	B	Yes	M184V		
	BAS053	B	Yes	M184V		
	**BAS065** ^***a***^	**B**	**Yes**	**M41L, L210W, T215Y**	**Y181C**	**M46L, I54V, V82A**
	**BAS069** ^***a***^	**BFi**	**Yes**	**K70R, M184V**	**L100I, K103E**	**I54V, L90M**
	BAS075	BFi	Yes		K103N	
	**BAS087** ^***a***^	**B**	**Yes**	**M41L, L210W, T215Y**	**L100I, K103N**	**M46L, I54V, V82A**
	BAS095	B	Yes	M41L, M184V, T215Y	K103S, G190A	
	BAS102	B	Yes	M41L, M184V, T215Y	Y188L	
	**BAS111** ^***a***^	**B**	**Yes**	**K70R, T215F**	**Y188L**	**I50V, I54L, V82A, L90M**
	BAS113	B	NA		Y181C	
	BAS134	B	Yes	M41L, K70R, M184V, L210W, T215Y		
	BAS141	B	Yes	M41L, M184V, L210W		
	BA25MLS^b^	B	No		K103N	
	BA 91ISM^b^	B	No			M46I, V82T
	BA111EGS^b^	BFi	No		G190A	
	BA114EGM^b^	B	No			L90M
	BA115MSP^b^	B	No	K65N		
FSA	BR100FSM145	B	Yes	K70R		
	BR145FSC100	B	Yes	M41L, M184V	G190S	
	BR39FS	B	Yes		A98G	
	BR47FS	B	Yes	K70R	K103N, Y181C, G190A	
	BR49FS	F	Yes	M41L, M184V	K101E, G190A	
	BR51FS	B	Yes			M46I, I50L, V82A
	BR52FS	F	Yes	M41L, M184V	A98G	
	BR57FS	B	Yes	M41L, T69D		
	BR60FS	B	Yes	M184V	K103N	
	BR85FS	B	Yes		L100V	
	BR91FSM83	B	Yes	K70R, M184V		I54L, N88T

a: individuals with NRTI, NNRTI and PI resistance; b: individuals drug naïve; FSA: Feira de Santana city; NA: not available; NNRTI: non-nucleoside reverse transcriptase inhibitors; NRTI: nucleoside reverse transcriptase inhibitors; PI: protease inhibitor; SSA: Salvador city.

There was no statistically significant relationship between mutations and age, however it is important to note that the individual BR145FSC100 is a 9-year-old child (Table IV) with the mutations M41L, M184V e G190S, which in combination confer high levels of resistance to the drugs 3TC and FTC, intermediate levels of resistance to d4T and Didanosine (DDI) (in the NRTI group), and high levels of resistance to EFV and NVP (in the NNRTI group).

Out of the 30 individuals with high and intermediate ARV resistance mutations, five (16.7%) were drug-naïve. For all classes of ARVs, the prevalence of mutations conferring intermediate and high resistance levels were higher among patients under treatment (26.1%) than among drug-naïve individuals (4.2%) (p ≤ 0.001). Among 92 treated individuals that were characterised by genome sequences of the *pol* region, six (6.5%) had mutations conferring resistance against PIs, 19 (20.6%) against NRTIs, and 15 (16.3%) against NNRTIs. Among 118 drug-naïve individuals with resistance mutations, two (1.7%) had mutations conferring resistance against PIs, one (0.9%) against NRTIs, and two (1.7%) against NNRTIs.

## DISCUSSION

Brazil is a large country with diverse HIV-1 genotype patterns in its geographic regions. Bahia is the most populous state in the North-east Region, with the highest number of AIDS/HIV cases (Monteiro et al. 2009). The clinical, molecular, and epidemiological characterisation of the HIV-1-seropositive population is important to better understand the local epidemic. In this report, the *gag*, *pol*, and *env* sequences for each individual genomic region that had been obtained in previous studies were combined, allowing for the determination of a more reliable genotypic profile of the HIV-1 variants circulating in this area. As determined by these previous studies, and confirmed in the present study, it is clear that the B genotype is still predominant in Bahia (Monteiro et al. 2009, Santos et al. 2009, 2011, Araújo et al. 2010, Monteiro-Cunha et al. 2011).

The present study demonstrated that the age group with the highest HIV infection rate was 31-40 years. The epidemiological profile chart resembles a pyramid (Fig. 2A), which verifies the pattern described by the Ministry of Health for the Brazilian infected population (MS 2015) and by the Bahia Secretariat of Health Surveillance (SOHS) for Bahia state population (SUVISA 2015), both of which reported the majority of cases occurring in the age range of 31-40 years. It has previously been asserted that HIV is more prevalent in men than in women (MS 2015). Conversely, we determined that most HIV infections occur in females, with 50.2% of the population (ratio 0.92:1) infected. However, a cohort that only evaluates the mother-to-child transmission, with 50 women and eight children (Monteiro-Cunha et al. 2011), was included in our meta-analysis and we thus excluded this cohort for gender analysis calculations. Among the remaining 205 patients, 122 (59.5%) were men. The male to female ratio in this group was 1.47:1, similar to that reported by the SOHS for the entire Bahia population, which was reported to be 1.5:1 between 2002 and 2012 (SUVISA 2015).

The highest risk of exposure in this cohort was heterosexual contact (40.7%), followed by MSM (19.0%) (Fig. 2B). The AIDS bulletin published by the SOHS reported that both heterosexual and homosexual contact were responsible for viral transmission (61.0% and 15.8% respectively) in the Bahia population from 2005 to 2014 (SUVISA 2015). The differences between these rates and the rates observed in the present study might be because the exposure category was not informed by almost 30% of the population included in the present analysis (Table I). In addition, the results presented here may be biased owing to the fact that we included a cohort of women and children infected by vertical transmission. In fact, in the present study, a rate of 4.56% of vertical transmission was observed, whereas the data reported by the Secretariat indicates a rate of 0.8% in the period from 2005 to 2014.

The subtype B male/female ratio remains higher for men (1.23:1), and the opposite occurs for F subtype-related viruses (0.46:1). Furthermore, 76.8% of the individuals infected with either F or BF genotypes in this analysis were infected through heterosexual transmission. From another point of view, when the entirety of heterosexually infected individuals were analysed, we found that 40.2% carry the F or BF virus, whereas among individuals infected through another transmission path, only 16.7% carry the F or BF viral subtype (p < 0.001). On the other hand, among individuals infected through homosexual contact, subtype B is predominant (86%) (p < 0.01). These results indicate an association between heterosexual contact and the F viral subtype, whereas the transmission of the B subtype is associated with homosexual contact.

For all classes of drugs analysed, the prevalence of mutations associated with therapeutic resistance was higher among individuals under treatment (6.5% PIs, 20.65% NRTIs, NNRTIs 16.3%) than among the drug-naïve (PIs 1.7%, 0.9% NRTIs, NNRTIs 1.7%). Brites et al. (2016) defined the mutational profile associated with ARV resistance in infected individuals from five different cities in Brazil, and concluded that DRV and TPV are the most susceptible drugs in the PI group in Salvador city, which was confirmed in the present study. We also verified that there was lower susceptibility to 3TC in the NRTI group, and to NVP and EFV in the NRTI group (Brites et al. 2016), with 3TC and EFV in combination with TDF used as the first line of treatment (IST-AIDS Hepatites Virais 2013). Clinical relevance was also demonstrated for the individuals BAS065, BAS069, BAS087, and BAS111 (Table IV), who had high and intermediate levels of resistance to all drug classes (PI, NRTI and NNRTI) while under treatment. These observations reinforce the idea that ARV treatment may lead to eventual drug failure, once exposure drives high selective pressure to the acquisition of resistance (Brites et al. 2016).

We found a significantly lower prevalence (4.2%) of transmitted drug resistance (TDR) within the drug-naïve population during the 2006-2012 period, compared with previous studies concerning drug-naïve individuals in Bahia (Table III). Pedroso et al. (2007) reported 18.9% TDR, whereas Soares et al. (2014) reported 17% TDR (p ≤ 0.01). The prevalence of resistance to NRTIs was lowest in the untreated population (0.9%), which could perhaps indicate a low TDR. The NRTI resistance in this dataset was markedly lower than that observed by Pedroso et al. (2007) (9.8%) and Soares et al. (2014) (10.6%). On the other hand, the prevalence of drug resistance among ARV-treated individuals (26.09%) was similar to that found by Monteiro-Cunha et al. (2011) (17.2%) in Feira de Santana and by Santos et al. (2011) (24.6%) in Salvador. This similarity is expected, since the current study includes samples from both previous studies, in which 82.8% and 77.2% of the analysed population were receiving antiretroviral treatment, respectively. The low prevalence of drug resistance among treated individuals might be related to the genomic origin of the analysed sequences. Significantly fewer mutations have been found in proviral DNA compared to historic viral RNA genotypes, which suggests that some resistance mutations are not archived or are not detectable in the latent reservoir (Wirden et al. 2011). Moreover, out of 92 treated individuals, 42 (45.7%) had undetectable viral load, indicating that these individuals were not failing therapy. This observation, in addition to other unidentified inclusion bias, such as sample collection during treatment suppression and very low treatment adherence, could also contribute to the low resistance rate observed within this group. Among ARV-treated individuals, the NRTI resistance rate was always higher than that of other drug classes (Monteiro-Cunha et al. 2011, Santos et al. 2011), which was similarly observed in the drug-naïve cohort studied by Soares et al. (2014). Overall, these observations point to the need for better designed population studies to accurately determine the drug resistance prevalence in this area.

Only four (16.7%) out of 24 individuals with drug-resistance mutations had information regarding their drug regimen. BAS015 was being treated with AZT+3TC+IDV and had K70R and M184V mutations, which confer resistance to both AZT and 3TC. However, this patient presented viral suppression with undetectable viral load, which indicate the infection was under control by IDV. BA065 was being treated with AZT+3TC+EFV and had mutations (Table IV) that confer resistance to all three drugs included in their drug regimen, suggesting therapy failure. In fact, this patient had experienced virologic failure (3.4 × 10^5^ copies/mL). BA075 was being treated with AZT+RTV and presented the K103N mutation, which indicated that they had either been re-infected with a resistant variant or experienced a previous treatment combination. This patient had a high viral load (7.3 × 10^5^ copies/mL). BAS134 was being treated with D4T+ATV and had mutations (Table IV) that confer resistance to D4T but not to ATV. Nonetheless, this patient had a viral load of 9.1 × 10^3^ copies/mL. Overall, these findings reinforce the importance of HIV genotyping for better clinical management of patients experiencing therapy failure.

The HIV epidemic in Bahia state has a high diversity of viral genotypes, but subtype B has the highest prevalence in the population as well as in the ARV-resistant population. This work highlights the importance of characterising the population ARV drug-resistance profile, highlighting important clinical cases. Through this report, it will be possible to better understand how HIV is circulating in the local population and thus develop new strategies to better manage the epidemic.
